# How I treat obesity and obesity related surgery in patients with chronic myeloid leukemia: An outcome of an ELN project

**DOI:** 10.1002/ccr3.3738

**Published:** 2021-01-08

**Authors:** Mohamed A. Yassin, Nancy Kassem, Rola Ghassoub

**Affiliations:** ^1^ Hematology Section Medical Oncology National Center for Cancer care and Research HMC Doha Qatar; ^2^ Department of Pharmacy National center for cancer care and Research HMC Doha Qatar

**Keywords:** chronic myeloid leukemia, obesity, surgery, TKIs level, tyrosine kinase inhibitors

## Abstract

Obesity may affect treatment outcome in CML patients, therefore the treatment of this cohort of patients need careful monitoring, TKIs dose adjustment may be required for certain patients. Further studies are needed to determine the proper TKIs doses.

## Graphical abstract

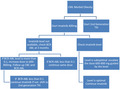

## 1 INTRODUCTION

Obesity is a chronic disease that is increasing in prevalence in adults, adolescents, and children and it is now considered to be a global epidemic. Current recommendation for treatment of Chronic Myeloid Leukemia (CML) does not take in consideration the weight of the patient with regard to doses of different Tyrosine Kinase Inhibitors. Obesity and obesity related surgeries are emerging and unmet needs. To shed the light into this special category of patients and we provide a strategy to treat morbid obesity with CML as well as obesity related surgeries like gastric band, sleeve gastrectomy in patients with CML. Five case scenarios were used to illustrate what is seen in real life: CML (CP) with class III obesity, CML (CP) with sleeve Gastrectomy, CML (CP) post gastric band CML with blast crisis each scenario were followed by proposal for treatment. Obesity and obesity related surgeries are emerging and unmet needs, the treatment of each should be individualized, and the TKI dose may needs to be adjusted in certain categories.

The morbidity and mortality associated with being overweight (body mass index [BMI] of 25‐29.9 kg/m^2^) or obese (BMI of ≥ 30 kg/m^2^) have been known to the medical profession for more than 2000 years. Obesity is a chronic disease that is increasing in prevalence in adults, adolescents, and children and it is now considered to be a global epidemic.^1^ Body mass index (BMI) based classifications were originally based upon the associated risks of cardiovascular disease (CVD). The recommended classifications for BMI, adopted by the National Institutes of Health (NIH) and World Health Organization (WHO),^2^, ^3^ for Caucasian, Hispanic, and black individuals are:

Underweight – <18.5 kg/m^2^
Normal weight – ≥18.5 to 24.9 kg/m^2^
Overweight – ≥25.0 to 29.9 kg/m^2^
Obesity – ≥30 kg/m^2^
Class I – 30.0‐34.9 kg/m^2^
Class II – 35.0‐39.9 kg/m^2^
Class III – ≥40 kg/m^2^ (also referred to as severe, extreme, or massive obesity)

Chronic Myeloid Leukemia (CML) is a clonal disorder of multipotent hematopoietic stem cells that accounts for 10% of all leukemias. The Philadelphia chromosome, a reciprocal translocation between chromosomes 9 and 22, t(9; 22)(q34; q11.2), is the hallmark of this rare leukemia.^4^ Little is known about the clinical features that may predispose or be linked to CML etiology. Several risk factors have been evaluated in a few studies with mixed results. Exposure to high‐dose radiation is the only established risk factor thus far.^5^

Smoking, pesticides, benzene/solvents, hair dye, and extremely low‐frequency electromagnetic fields have been postulated as potential risk factors, but the results are inconclusive.^6^ Accumulating evidence suggests that obesity may increase the risk of several adult solid tumors, as well as adult hematopoietic cancers.^7^, ^8^ Obesity has reached epidemic proportions in the United States; determining the role that this modifiable condition plays in the risk of many different types of cancer is a significant public health issue. Current recommendations by the European Leukemia Net (ELN) for CML does not take in consideration the weight of patients with regard to doses of different Tyrosine Kinase Inhibitors (TKIs).^9^ Obesity and obesity related surgeries represent emerging and unmet needs in the management of CML. There is very limited data with regards to the effect of obesity and gastric surgeries on CML treatment. Recently, it has been suggested that assessment of BMI at baseline should be considered as a decision factor when starting treatment in CML patients.^10^, ^11^ In a cohort study including 78 patients with chronic phase CML treated upfront with second‐generation tyrosine kinase inhibitors (TKIs) according to BMI at diagnosis, a significant difference in molecular response was reported between patients with low (<25) and high (>25) BMI who received nilotinib or dasatinib as frontline treatment.^11^

## 2 SPECIFIC PATIENTS

### 2.1 Morbid obesity and imatinib

#### 2.1.1 Case 1

A 27‐year‐old morbidly obese female, (weight of 275 kg; height 156 cm and BMI 113 kg/m^2^) was found to have leukocytosis on a routine checkup. The patient's work up, including peripheral smear, fluorescence in situ hybridization (FISH) and conventional cytogenetics confirmed CML in chronic phase (CP). Subsequently, the patient was started on Imatinib 400 mg once daily. At 3 months, she achieved complete hematological remission but she did not achieve the required molecular target. A mutation analysis was performed to rule out an imatinib resistant primary mutation but was negative.

Due to the lack of evidence on how to treat such obese patients, the options were to switch to a second‐generation tyrosine kinase inhibitor or to escalate the dose of imatinib, with close observation. The patient opted for the latter and so the imatinib dose was increased to 400 mg twice daily. The patient remained in hematological remission and by her next 3‐month assessment she also showed a Major Molecular Response (MMR) which was subsequently maintained.

##### Recommendations

**FIGURE 1:**
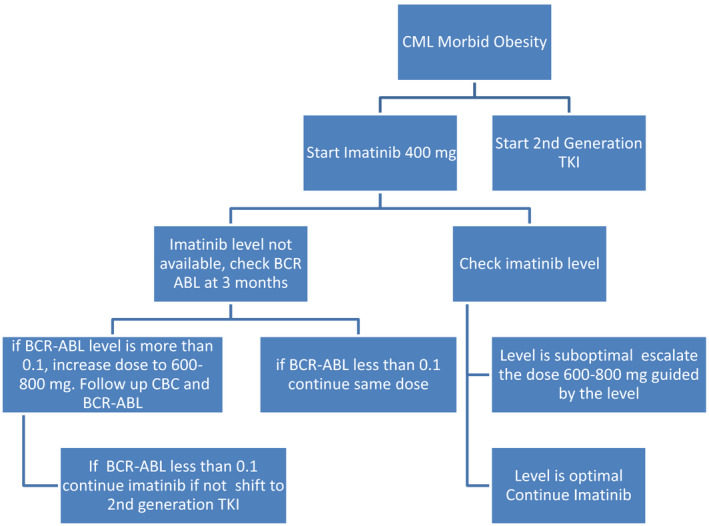
Imatinib as upfront for Morbid Obese patient with CML

Morbidly obese CML chronic phase patients starting TKIs as upfront therapy may require higher doses of Imatinib or to start with a second‐generation TKI and wait a longer duration for response Figure 1.

### 2.2 Imatinib post sleeve gastrectomy

#### 2.2.1 Cases 2 and 3

Yassin et al reported two cases of morbid obesity with CML, one on dasatinib and the other on nilotinib, who underwent sleeve gastrectomy and had subsequent loss of hematological remission. Both patients were switched to Imatinib and subsequently achieved complete hematological remission (CHR), complete cytogenetic remission (CCyR) and a major molecular response (MMR).^12^

##### Recommendations

**FIGURE 2:**
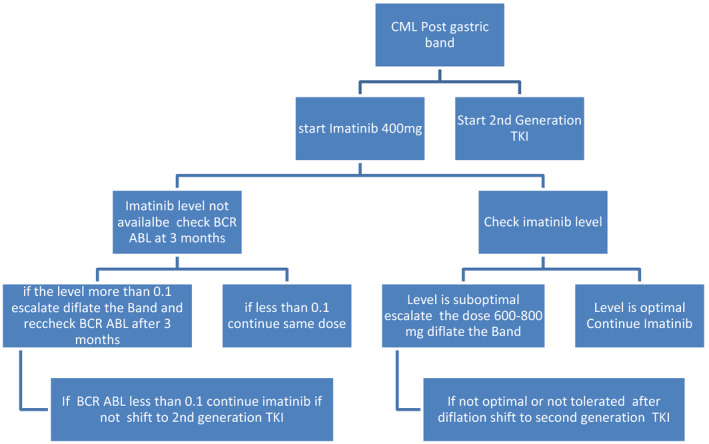
Imatinib post sleeve Gastrectomy

Patients with CML on second‐generation TKIs who underwent sleeve gastrectomy and lost their response can benefit from switching to Imatinib See Figure 2.

### 2.3 Morbid obesity and gastric band surgery

#### 2.3.1 Case 4

A 22‐year‐old man, morbidly obese, with gastric band, his weight 166 kg, height 180 cm and BMI was 51.23 kg/m^2^, was newly diagnosed with CML in chronic phase and started on imatinib 400 mg daily. He was considering having a sleeve gastrectomy and seeking medical advice about whether he can go for such procedure with the current diagnosis or to remove the gastric band.

Sleeve gastrectomy was not recommended due to the potential for a drastic change in drug absorption, which could affect treatment efficacy. An alternative option for this patient was to deflate the band. Before deflation, the patient achieved CHR but did not reach the molecular target at 3 months. After the deflation procedure, the patient achieved the molecular target.

##### Recommendations

In patients with morbid obesity, bariatric surgeries like sleeve gastrectomy and gastric bypass are not recommended.

**FIGURE 3:**
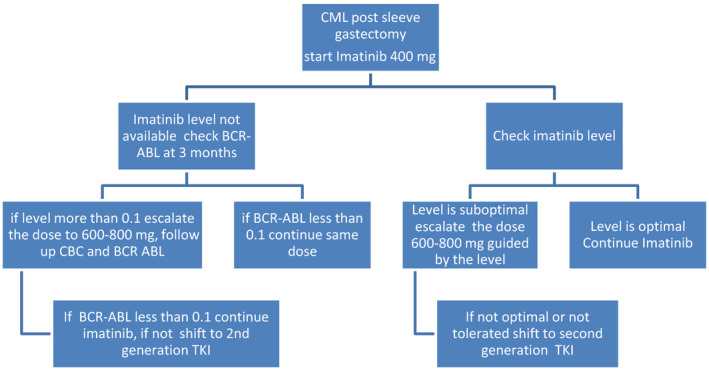
TKIs post gastric band

For patients with gastric band, the band should be removed or deflated to allow normal gastric transit time and not to interfere with drug absorption See Figure 3.

#### 2.3.2 Case 5: progression to blast crisis

A 30‐year‐old morbidly obese man underwent sleeve gastrectomy. Two years later, he was diagnosed with CML chronic phase and was started on Nilotinib as upfront therapy. The patient achieved complete hematologic remission (CHR) but not a major molecular remission (MMR). Therefore, he was switched to imatinib. There had a subsequent 2 log reduction in BCR‐ABL but he did not achieve the necessary target by 3 months.

Pegylated interferon alpha‐2a at 135 µg subcutaneously once per week was added to the imatinib but despite this he progressed to myeloid blast crisis.

##### Recommendations

Patients with sleeve gastrectomy who are not responding to one or more tyrosine kinase inhibitor should start an early donor search for possible allogenic stem cell transplantation.

## 3 DISCUSSION

The prevalence of obesity is increasing rapidly; it is well known that obesity can affect different organs in the human body such as the liver and may affect some physiological processes such as cardiac output, gastric emptying and gut permeability.^13^, ^14^, ^15^ However, the impact of this effect on drug absorption and metabolism remains unclear. The dosing of medications is still a major challenge for clinicians who treat the overweight population.

Very few studies have addressed the impact of obesity on pharmacokinetics and pharmacodynamics of drugs or described alterations of drug metabolism and clearance in obese patients.^16^, ^17^, ^18^ it is important to note that CYP3A4 is the major enzyme responsible for metabolism of TKIs; the main circulating active metabolite of imatinib is the N‐demethylated piperazine derivative, formed predominantly by CYP3A4. It shows in vitro potency similar to the parent imatinib. The plasma AUC for this metabolite is about 15% of the AUC for imatinib.^18^ Reduced CYP3A4 metabolic activity and consequently decreased clearance of some medications has been reported in obese patients. Carbamazepine clearance was slightly reduced in obese compared to nonobese patients; on the other hand, its clearance significantly increased after major weight loss in some of the obese patients.^19^, ^20^ The suggested explanation for this was the presence of fatty liver that disappeared after weight loss. This fatty liver might have affected the drug metabolism through inhibition of some biochemical reactions in the liver or by reducing the liver blood flow.

Furthermore, increased gut wall permeability and gastric emptying were observed in obese individuals, this in turn is believed to affect the oral absorption of drugs and nutrients.^21^, ^22^, ^23^

If we consider these observations in relation to the first patient, alteration of imatinib absorption and/or metabolism to the active metabolite may have resulted in reduced exposure to the drug. Increasing the dose above the conventional recommended dose (to 600 or 800 mg) may well overcome this phenomenon, as seen in the first patient who achieved a major molecular response following dose escalation.

In 2012, Breccia et al reported a longer median time to achieve complete cytogenetic response (CCyR) (68 months vs 33 months, *P* = .001) in patients with a BMI > 25 kg/m^2^ who had received imatinib as upfront therapy. The study also reported a reduced rate of major molecular response (MMR) (77% vs 58%, *P* = .001), which was also achieved over a longer median time (29 months vs 14 months, *P* = .001).^9^ Conversely, no significant differences were observed in achieving molecular response with respect to BMI in patients treated frontline with the second‐generation TKIs, nilotinib and dasatinib.^10^

These findings suggest that obese patients may respond better to second‐generation TKIs.

Obese patients who undergo bariatric surgery may have different behavior with regards to drug absorption and metabolism and the differences can vary depending on the type of surgery or procedure (Table 1).^24^, ^25^, ^26^, ^27^, ^28^, ^29^ see Table 1

**TABLE 1:** The most common bariatric surgeries and their effects on drug absorption

Procedure	Description	Possible effects on absorption
Laparoscopic adjustable gastric banding	A synthetic band placed distal to the gastroesophageal junction to create a gastric pouch. This procedure limits the volume of the proximal stomach and therefore restriction of oral intake.	Might affect the volume and transit time, which may alter the pharmacokinetics of medications.^24^
Laparoscopic sleeve gastrectomy	A partial gastrectomy removing the greater curvature creating a tubular gastric conduit.	Reduces gastric volume, increases gastric PH, accelerates gastric emptying ^25^
Roux‐en‐Y gastric bypass	Creating a small gastric pouch in the upper fundus of stomach, which is then connected to the jejunum, bypassing most of the stomach and upper small intestine	Reduces gastric volume, increases gastric PH, delays gastric emptying, decreases surface area of absorption^29^

Studies have reported an increased activity of CYP3A4 metabolism after gastric bypass surgery, resulting in increased clearance of drugs such as cyclosporin, tacrolimus, sirolimus and mycophenolate.^25^, ^26^

Bariatric surgery can also affect absorption of nutrients and orally administered drugs; this can be attributed to reduced absorptive surface, bypassing the acidic environment of the stomach, changes in gastric emptying rate, or changes in the active transport mechanisms. The major causes of decreased absorption in patients post bariatric surgery are thought to be the reduced gastrointestinal surface area and the increased gastric pH. Drugs that are soluble in an acid environment are more likely to be absorbed in the stomach and it is these medications that are likely to be affected in patients who undergo gastric bypass surgery or sleeve gastrectomy as the gastric acid production is decreased, leading to decreased solubility and absorption of acid‐soluble drugs.^27^, ^28^, ^29^

Studies describing the pharmacokinetics and pharmacodynamics of TKIs in obese patients or patients who underwent bariatric surgeries especially in CML patients are limited to two case reports.^30^, ^31^

Most TKIs exhibit pH‐dependent solubility; imatinib, nilotinib and dasatinib are soluble in an acidic pH. Imatinib and nilotinib solubility rapidly declines above pH 5.5 and 4.5 respectively (Table 2).^32^, ^33^

**TABLE 2:** TKIs solubility

Drug	Solubility
Imatinib	Soluble in aqueous buffers ≤ 5.5 Very slightly soluble to insoluble in neutral/alkaline aqueous buffers.^32^
Nilotinib	Solubility strongly decreases with increasing pH, and is practically insoluble in buffer solutions of pH ≥ 4.5.^33^
Dasatinib	Exhibits pH‐dependent aqueous solubility (from 18.4 mg/ml at pH 2.6‐0.008 mg/mL at pH 6.0).^34^

The co‐administration of dasatinib with gastric acid reducing agents can significantly decrease the concentrations of dasatinib with reduced mean AUC of dasatinib by up to 61% and the mean Cmax of dasatinib by up to 63%.^34^ see Table 2.

One of the most common complications of sleeve gastrectomy is reflux esophagitis. All patients post sleeve gastrectomy are required to be on proton pump inhibitors at least for 6 months. This would increase the gastric pH, which can explain the loss of effect of dasatinib post sleeve gastrectomy in case 2.

It is known that acidity of the gastrointestinal tract varies through different segments. It declines from the stomach (pH 1‐3) to the intestines (pH 5‐8),^35^ hence the solubility and absorption of those TKIs are thought to occur mainly in the stomach and declines through the gastrointestinal tract.

Two studies addressed the impact of gastrectomy on both imatinib and nilotinib exposure in gastrointestinal stromal tumors (GIST) patients. The first study revealed significantly lower imatinib trough levels in patients with previous gastrectomy compared to patients without gastric surgery. Similarly, the second study showed decreased plasma drug exposure and bioavailability of nilotinib in patients with gastrectomy.^36^, ^37^

In another study involving sunitinib, there was no impact of major gastrectomy on sunitinib plasma exposure although its solubility is known to be maintained even up to a higher pH of more than 6.8. The study suggested that the effect of gastrectomy on drug exposure might vary from one TKI to the other depending on their physiochemical properties and their absorption characteristics throughout the gastrointestinal tract, in addition to the resected part of gastrointestinal tract.^38^

It is therefore suggested that in patients with sleeve gastrectomy or gastric bypass, reduced gastric acid secretion may contribute to a decreased solubility and absorption and consequently decreased effect of TKIs as seen in cases 2, 3 and 5.

Although this hypothesis has not been addressed in randomized trials, two case reports have described the effect of bariatric surgeries on TKIs in CML patients.

One case report described a morbidly obese CML patient who achieved complete molecular response with imatinib 400 mg/day and after four years she underwent sleeve gastrectomy. Imatinib trough concentrations were compared before and after surgery and were found to be reduced by 46%‐60% after surgery. ^30^

Another case reported an obese patient with CML who had a biliopancreatic diversion with duodenal switch for weight loss; this patent was treated with imatinib 400 mg/day before the gastric bypass surgery and had complete hematological remission (CHR), and complete cytogenetics remission (CCyR) and good molecule response with a BCR‐ABL/c‐ABL ratio of 0.015 (1.5%). After the surgery, she was found to have a decline in imatinib trough level to 17% of the presurgery level and consequently had a 0.5 log increase in BCR‐ABL/c‐ABL ratio of 0.083 (8.3%). The imatinib trough level increased again to a level close to therapeutic level after escalating the dose to 400 mg BID; however, after the patient lost significant weight, the imatinib level dramatically increased and it was suggested that a correlation exists between imatinib dose and the actual body weight. ^31^

The Laparoscopic Adjustable Gastric Banding (LAGB) technique is based on a synthetic band placed distal to the gastroesophageal junction to create a gastric pouch. This procedure limits the volume of the proximal stomach and therefore restriction of oral intake.^24^ this in turn will affect the gastric volume and transit time, which may alter the pharmacokinetics of the TKIs. This is why our patient in case 4 was advised to remove the band or to deflate it, with a good outcome.

With regard to drug levels monitoring our recommendation will be as illustrated in pharmacotherapy study by Miura“For patients not achieving a satisfactory response within 3 months, the mean plasma concentration for the three months of TKI administration must be considered. In TKI therapy for CML patients, therapeutic drug monitoring is a new strategy for dosage optimization to obtain a faster and more effective clinical response. The imatinib plasma trough concentration (*C*₀) should be set above 1000 ng/mL to obtain a response and below 3000 ng/mL to avoid serious adverse events such as neutropenia. For patients with a UGT1A1*6/*6, *6/*28, or *28/*28 genotype initially administered 300‐400 mg/day, a target nilotinib *C*₀ of 500 ng/mL is recommended to prevent elevation of bilirubin levels, whereas for patients with the UGT1A1*1 allele initially administered 600 mg/day, a target nilotinib *C*₀ of 800 ng/mL is recommended. For dasatinib, it is recommended that a higher *C*_max_ or *C*₂ (above 50 ng/mL) to obtain a clinical response and a lower *C*₀ (less than 2.5 ng/mL) to avoid pleural effusion be maintained by once daily administration of dasatinib. Although at present clinicians consider the next pharmacotherapy from clinical responses (efficacy/toxicity) obtained by a fixed dosage of TKI, the TKI dosage should be adjusted based on target plasma concentrations to maximize the efficacy and to minimize the incidence of adverse events “.^39^

## 4 CONCLUSION

Obesity and obesity related surgeries may affect treatment outcome in CML patients, therefore the treatment of this cohort of patients need careful monitoring, TKIs dose adjustment may be required for certain patients. Further studies are needed to determine the proper TKIs doses for those patients.

## CONFLICT OF INTEREST

Authors declare no competing interests.

## AUTHORS' CONTRIBUTIONS

Yassin MA concept writing editing Final approval of manuscript. NK writing editing and final approval of the manuscript. RG editing and final approval of the manuscript.

## ETHICS APPROVAL

Ethics approval obtained from Hamad medical corporation IRB No MRC 01‐18‐337 and consents to participate were obtained from patients including consenting for publication.

## Data Availability

I confirm Availability of data and material.
